# Validation of Salivary Markers, IL-1β, IL-8 and Lgals3bp for Detection of Oral Squamous Cell Carcinoma in an Indian Population

**DOI:** 10.1038/s41598-020-64494-3

**Published:** 2020-04-30

**Authors:** Prerana Singh, Jitendra K. Verma, Jayant Kumar Singh

**Affiliations:** 1 Department of Oral Pathology, Maharana Pratap Dental College, Kanpur, UP India; 2 Department of Radiotherapy, J K Cancer Institute, Kanpur, UP India; 3 Department of Chemical Engineering, Indian Institute of Technology Kanpur, Kanpur, UP India

**Keywords:** Diagnostic markers, Oral cancer detection

## Abstract

Early detection and easier follow-up of oral squamous cell carcinoma (OSCC) would significantly improve the morbidity and mortality associated with it. With newer technologies, it has become possible to validate cancer biomarkers in saliva with high sensitivity and specificity. There is however a need to further validate these biomarkers in cohorts of different ethnic groups. Our objective was to validate previously evaluated salivary biomarkers in Indian population. The study enrolled 117 patients. These were grouped into subcatergories of 31 early (TNMstage I-II) and 27 late-stage OSCC (TNM stage III-IV), 30 PMOD and 29 post-treatment patients. There were 42 control subjects. We evaluated 3 protein markers, IL-1β, IL-8 and LGALS3BP using ELISA, from unstimulated saliva samples. Statistical analysis was done to calculate p-value, ROC, AUC, sensitivity, and specificity. Protein markers IL-1β and IL-8 were significantly elevated (p < 0.05) in OSCC patients. Though the markers could not discriminate PMOD and post-treatment subjects from controls, they proved to be significantly discriminatory between OSCC and controls. Both these markers were especially strong discriminators of late stage OSCC (stage III-IV). IL-1β had the most statistically significant discriminative power (AUC = 0.9017) in late-stage OSCC followed by IL-8 (AUC = 0.7619). Although LGALS3BP was not found to be significantly elevated in late stage OSCC patients, but it was a significant discriminator of early stage OSCC (stage I-II) with p-value = 0.0008 and AUC = 0.7296. These salivary biomarkers have been discovered and validated in other ethnic groups earlier. Hence, the fact that these markers were discriminatory in Indian population too, strengthens the possibility of using these salivary biomarkers as screening tools in different ethnic cohorts. Such trials would potentiate use of a non-invasive tool, like saliva for diagnosis and follow-up of oral cancer.

## Introduction

Oral cancer has become a disease of concern worldwide with up-to 400000 new cases per year with almost 130000 deaths annually. Oral squamous cell carcinomas (OSCC) accounts for 90% of all oral cancers, of which 80% occur in Southeast Asia^1^. Oral cancer accounts for over thirty per cent of all cancers reported in the country. Hence, control and management of oral cancer has become a top priority in the health sector^2^. Early detection, mass-screening, and easy follow-ups would improve survival, and decrease mortality and morbidity associated with OSCC.

Though biopsy is the gold standard for diagnosis of OSCC, it is not convenient for screening and follow-up due to its invasive nature, high cost, and need for specially trained medical personnel and equipment. Moreover, the current tools of diagnosis are not enough for detecting high risk PMODs (potentially malignant oral disorders) or in post-treatment phases during follow-up, as DNA mutations have been observed even in epithelial cells with no evidence of histopathological changes. Thus it is of utmost importance to develop newer, non-invasive and easy to use diagnostic medium and tools for the detection of OSCC. The detection of discriminatory biomarkers in saliva samples is considered to be the most promising answer at this stage^3^.

The mutational events that leads to transformation of healthy cells into malignancy can cause altered expression of proteins and mRNA markers in saliva. Clinical significance of salivary biomarkers in various malignancies has been studied by several investigators in breast cancer^4,5^, ovarian cancer^6^, salivary gland tumors^7^, gastric cancer^8^ and pancreatic cancer^9^. Further studies have potentiated the significance of salivary biomarkers even for OSCC detection.

The quest to find salivary biomarkers specific to oral cancer was followed by several investigators. Katakura *et al*.^10^ examined the expression of four kinds of cytokines in saliva. The authors measured the levels of IL-1β, IL-6, IL-8 and osteopontin in whole saliva samples, which were found to be higher in patients with oral cancer than in healthy controls. Serum IL-6 levels proved to be a significant independent predictor of recurrence as well as poor survival in a longitudinal, prospective cohort study by Duffy *et al*.^11^.

While studying cytokines in saliva samples, there are chances that cytokine levels may be altered due to other oral diseases. Therefore, Cheng *et al*.^12^ compared IL-6 levels in oral cancer, oral lichen planus (OLP), and chronic periodontitis (CP) patients. They found that salivary IL‐6 levels were significantly higher in patients with OSCC than in patients with CP, OLP, and healthy controls. Salivary IL‐8 levels too were significantly higher in patients with OSCC than in patients with CP, but only marginally significantly higher than in healthy controls.

Other investigators used proteomic targets other than the cytokines as promising salivary biomarkers for oral cancer detection. Hu *et al*.^13^ conducted a shotgun proteome analysis of saliva from healthy and OSCC patients and suggested a proteome biomarker panel (MRP14, profilin, CD59, catalase and LGALS3BP) for oral cancer detection. Nagler *et al*.^14^ tested 19 tongue cancer patients, measuring the levels of 8 salivary markers. They found increased levels of carbonyls, LDH, MMP-9, Ki-67 and Cyclin-D1 and decreased levels of OGG1, phosphorylated SrC and Maspin. Korostoff *et al*.^15^ found elevated levels of IL-1α, IL-6, IL-8, VEGF-α and TNF-α in saliva from patients of endophytic tongue SCC and suggested that these biomarkers can be helpful in identifying the progression of TSCC from a high risk lesion to neoplasm, thus serving in cancer screening and early detection.

More recently a larger array of proteomic as well as transcriptomic biomarkers have been evaluated. One such attempt was made by Brinkmann *et al*.^16^ in Serbian population. All the protein biomarkers (IL-1β, IL-8 and LGALS3BP) and four transcriptomes (*IL-8, IL-1β, SAT1*, and *S100 P*) were significantly elevated (p < 0.05) in OSCC patients. This study evaluated the discriminatory power of salivary transcriptomic and protein biomarkers in distinguishing OSCC cases from controls and PMOD’s.

Another study was conducted in a Taiwanese population^17^. Seven transcriptomic markers (*IL-8, IL-1β, SAT1, OAZ1, DUSP1, S100P*, and *H3F3A*) and two protein markers (IL8 and IL1β) were evaluated. *DUSP1* was significantly lower, while *IL-8* and *IL-1β* were significantly higher in OSCC patients than in controls and PMOD patients. Salivary *IL8p* and *IL-1β* together were most discriminatory between OSCC patients and controls. In addition, *IL8p* and *H3F3A* mRNA together were discriminatory between OSCC and PMOD patients. Yu *et al*.^18^ further contributed by generating a candidate biomarker panel for Taiwanese population using CART analysis, simulations and logistic regression. Their four protein panel included MMP1, KNG1, ANAXA-2 (annexin) and HSPA-5 (heat shock protein) with reported sensitivity of 87.5% and specificity of 80.5%. The authors also reported follow-up results of PMOD patients, where 18/88 developed cancer, and of these, 14/18 PMOD patients progressing to cancer had a risk score >0.4. Therefore, according to their suggested panel OSCC1 and high risk PMOD patients had a risk score >0.4. Wu CC *et al*.^19^ validated another salivary biomarker, resistin (RETN) in a large cohort of Taiwanese patients.

Csősz É *et al*.^20^ investigated 14 proteins, IL-1α, IL-1β, IL-6, IL-8, TNF-α, VEGF, catalase, profiling-1, S100A9, CD59, M2BP, CD44, thioredoxin and keratin 19 in Hungarian population. Several rounds of optimization on SRM based method for rapid salivary protein detection (RAPD) was developed. Subsequently, validation by ELISA revealed that the salivary proteins S100A9 and IL-6 were useful in improving the diagnostic accuracy for OSCC.

While significant works have been reported in South Asian and European populations, not much has been studied on Indian populations. Punyani *et al*.^21^ in a recent work on Indian population found that salivary IL-8 can be utilised as a potential biomarker for OSCC. However, their study was non-conclusive for oral premalignancy due to limited sample size. Rajkumar *et al*.^22^ in a similar attempt collected saliva and blood samples from OSCC, PMOD, and healthy subjects. Their study confirms that salivary IL-8 can be discriminatory between PMOD and OSCC.

A proteomic investigation in India was recently done by Sivadasan *et al*.^23^ on saliva of healthy individuals. They provided an updated proteome profile of saliva and a priority list of 139 proteins that can serve as target salivary markers for detection of oral cancer. However, these target proteomic biomarkers have not been validated in OSCC patients, yet in Indian population.

Though not much work has been done on salivary proteome and transcriptome OSCC markers in Indian population, salivary metabolomes have been studied by several investigators^24^. Sainger *et al*.^25^ evaluated the role of *CYP1A1*, *GSTT1* and *GSTM1* gene polymorphism from saliva to understand their role in detection of oral cancer. Shivashankara and Prabhu^26^ reported elevated levels of total proteins, free sialic acid and protein-bound sialic acid in OSCC patients and inferred that glycoproteins may have a role in carcinogenesis. In addition, elevated malondialdehyde and decreased glutathione levels were indicative of oxidative stress in oral cancer. Vajaria *et al*.^27^ found that in PMOD and OSCC patients, serum and salivary TSA/TP ratios and α-L-fucosidase activity were significantly higher compared to the controls. The levels of aforementioned metabolomes were found to be higher even in controls with tobacco habits. At the same time, salivary levels were elevated with a higher magnitude than serum levels of these proteins. Dadhich *et al*.^28^ suggested that sialic acid can be used as a reliable biomarker as it showed gradually increasing levels in both serum and saliva, from control to PMOD to OSCC subjects. As reported by the above studies, salivary metabolomes have been reported to be useful in diagnosis and prognosis of oral cancer in Indian population.

Hence, the next natural step in biomarker investigations in India would be to identify and validate proteomic and transcriptomic target molecules for detection of OSCC in Indian population. Neera *et al*.^29^ in their review, mentioned that in a multifactorial disease like cancer, genetic alterations do not always correlate with the complete expression of the disorder. Changes in protein structure and their expression levels play an important role in tumour development and progression. Thus, undoubtedly proteins are attractive molecular targets as potential biomarkers due to the fact that they participate more actively in cellular activities than DNA and RNA. Therefore, for our study we chose from the list of protein biomarkers, which have been reported as strong target molecules till date in the literature. We narrowed down our list of protein biomarkers to IL-1β, IL-8 and LGALS3BP, as they fulfilled the statistical criteria of significant efficacy with relatively high sensitivity, specificity and AUC values as reported by Yakob *et al*.^30^ in their review.

These markers have also been previously evaluated in different populations. While several studies have found promising results with these biomarkers, other studies have failed to validate them. Hence, our aim is to evaluate if these previously reported protein markers discovered and validated in OSCC patients in American, Serbian, Taiwanese and Hungarian populations^16–20^, are valid in Indian population too. The reason Indian cancer patients may show variations other than the genetic differences are the varied and mixed habit patterns displayed in the Indian population. Not only do these patients display habits associated with tobacco (smoking as well as smokeless tobacco chewing), but also other irritants like betel nut (betel quid, gutkha, and panmasala) and lime, catechu, etc. In fact smokeless tobacco chewing is more prevalent than smoking. Often these habits are seen in combinations, thus displaying a mixed habit pattern.

In addition, we also compared the marker levels in PMOD and post-treatment cohorts along with early (stage I-II) and late (stage III-IV) stages of OSCC. This would help us to evaluate the efficacy of these protein markers in all stages of OSCC, PMODs and post treatment phases. The findings would suggest if they can be of help in screening and post-treatment follow-ups. To the best of our knowledge, this is the first study in the Indian population, where multiple protein markers are evaluated in a case-control study design, to validate and compare the efficacy of all the three protein molecules in different stages of OSCC. If a non-invasive medium like saliva can be harnessed to detect OSCC, we can come up with efficient screening tools for large population cohorts.

## Methods

### Patient selection

Patients were selected from the JK Cancer Institute, Kanpur and Maharana Pratap Dental College, Kanpur. A case control study design was adopted. Case group had a total of 117 subjects, which were further grouped into subcategories comprising of 31 cases of stage I-II OSCC, 27 cases of stage III-IV OSCC, 30 cases of PMODs and 29 cases of post-treatment cases. Of these follow-up cases 13 were receiving interventions in the form of adjuvant chemotherapy and 8 were observed with recurrence. Control group had 42 subjects that were matched according to age, sex and socioeconomic status to each subcategory. All the subjects signed an informed consent approved by the ethics committee of institutions’ review boards. All methods were performed in accordance to the relevant guidelines and regulations of the Ethics Committee of JK Cancer Institute, GSVM, Kanpur. (EC Re-registration no. ECR/602/Inst/UP/2014/RR-17).

The diagnosis of case subjects was done after a thorough clinical examination and confirmed after a biopsy. American Joint Committee of Cancer (AJCC) system of TNM staging was followed. TNM (Tumour, Nodes, and Metastasis) staging evaluates the status of tumour size and extent of involvement, regional metastasis or nodal involvement, and distant metastasis. According to these parameters, the OSCC patients were broadly categorised into early (stage I and II) and late (stage III and IV) stages. PMODs involved high risk premalignant lesions of which maximum subjects (80%) had leukoplakia, and the rest had oral submucous fibrosis and oral lichen planus, with varying degrees of dysplasia on histopathology. The third subcategory of post-treatment cases included postoperative OSCC cases on their follow-ups, to evaluate if the levels of biomarkers can be of any indication of their disease status or recurrence. Thirteen (44.8%) of these follow-up postoperative cases OSCC patients were receiving interventions in the form of chemotherapy. Of these 8 patients had recurrent lesions.

Levels of cytokines alter depending on oral health and infection. Hence, to quantify whether the selected subjects were free of any oral inflammatory conditions, patients were examined clinically for any signs of inflammation and a complete blood profile was done. Liver and kidney function tests, and tests for diabetes and HIV were done to rule out immunocompromised status. Subjects that did not present with any clinical sign of inflammation and showed no signs of diminished immune status in the blood profile were chosen in both case and control cohorts. The inclusion and exclusion criteria used to select subjects are included in the supplementary section. (Supplementary Table S1)

The mean age of case subjects was 45.89 ± 12.36 yrs. The males were 81.2%. The tobacco chewers were 51.28%, and 31.62% had mixed tobacco habits (gutkha, tobacco chewing, and bidi smoking). The mean age for males and females was 46.15 ± 11.67 yrs and 45.73 ± 15.32 yrs, respectively. The mean age of the control subjects was 43.05 ± 10.40 yrs. The males were 80.95%, and 28.57% were smokers and/or chewers. The mean age for males and females was 43.18 ± 11.02 yrs and 42.63 ± 8.31 yrs, respectively. The epidemiological data is included in the supplementary section. (Supplementary Tables S2 and S3).

### Saliva collection and processing

Saliva samples were collected using Salivette^®^cortisol. Unstimulated morning saliva was collected from patients who had fasted one hour prior to collection. Saliva sample were freezed and stored at −80 °C. The thawed samples were centrifuged at 1000 × g at 2–8 °C for 20 min and then processed as per the guidelines of the ELISA kit from Elabscience to determine concentration of proteomic markers, IL-1β, IL-8 and LGALS3BP (galectin binding protein). All the samples were measured in duplicates and calculated with the respective standard curves. Transportation time between collection of samples and freezing ranged between 1 to 4 hours during which time sample was stored in a portable ice box. Storage time for any sample was not more than 2 months. Once thawed the samples were stored at -4 °C and used within a week.

### Statistical analysis

Sensitivity, specificity, receiver operating characteristic curve (ROC) and area under curve (AUC) were determined for each biomarker. Origin Pro 8 software was used for plotting graphs. Mann-Whitney U test was used for calculating the p-values, and Graph-Pad Prism 8.0 software was used for determining the sensitivity, specificity and ROC/AUC.

### Ethical approval

All the procedures were approved by the ethical committee of institutions’ review board.

## Results

We evaluated three salivary protein markers (IL-1β, IL-8, and LGALS3BP) in both case and control subjects. Case subjects included OSCC patients, PMODs and under-treatment cases. Of the three protein markers, IL-1β and IL-8 showed increased levels in OSCC patients compared to controls and therefore, could distinguish between cancer and control subjects as single markers but were strongly discriminatory, especially in case of stage III-IV as compared to control subjects, with p < 0.05. LGALS3BP showed increased levels in early stage OSCC cases and high risk PMODs (p < 0.05), as well as relatively decreased levels in late stage OSCC and patients undergoing chemotherapy or postoperative cases with recurrence.

**Figure 1:**
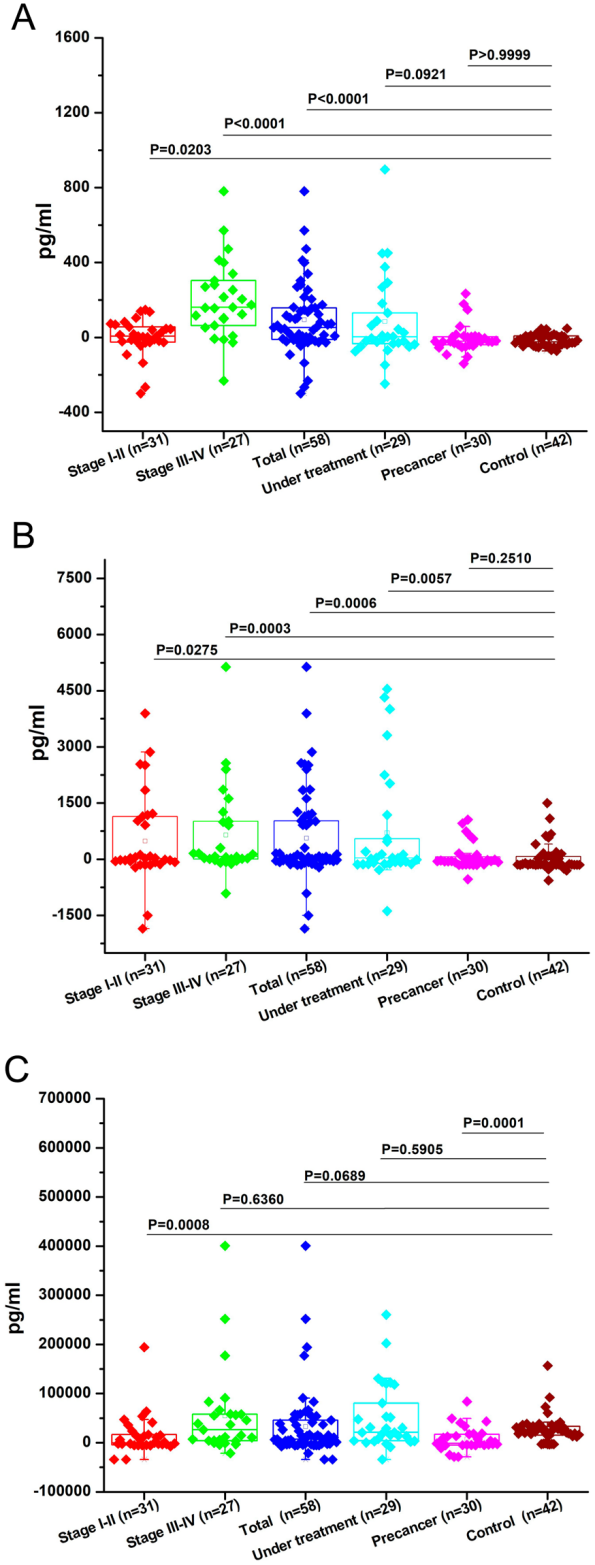
Salivary protein levels (mean and standard deviation) for IL-1β (panel A), IL-8 (panel B) and LGALS3BP (panel C); p-values with Mann-Whitney U test.

Figure 1 shows the concentration gradients of all three proteins in separate panels, A (IL-1β), B (IL-8), and C (LGALS3BP). Each panel shows salivary protein levels in OSCC stage I-II, OSCC stage III-IV, all OSCC cases together (OSCC total), PMODs, post-treatment stages and controls. Figure 1A shows IL-1β levels to be elevated in OSCC compared to controls, and post-treatment cases. In particular, levels were statistically significant for OSCC total (p < 0.0001), OSCC stage III-IV (p < 0.0001) and OSCC stage I-II (p = 0.0203). Figure 1B shows IL-8 levels to be elevated in OSCC and post-treatment cases compared to controls with statistically significant levels for OSCC total (p = 0.0006), OSCC stage III-IV (p = 0.0003), OSCC stage I-II (p = 0.0275)and post- treatment cases (p = 0.0057). Figure 1C shows LGALS3BP levels to be highly discriminatory for OSCC stage I-II (p = 0.0008) and PMODs (p = 0.0001). Hence, IL-1β was found to be a highly significant marker for late stage OSCC, whereas LGALS3BP was a found to be a highly significant marker for early stage OSCC and high risk PMODs. Also, IL-8 was found to be a good marker for all stages of OSCC and post-treatment cases.

Table 1 lists the p-values of the salivary biomarkers in each case group versus the control group. The available data suggests that IL-1β is a highly significant indicator in OSCC patients (p < 0.0001 in OSCC total and OSCC stage III-IV and p = 0.0203 in OSCC stage I-II) while IL-8 had reliable significance (p = 0.0006 in OSCC total, p = 0.0003 in OSCC stage III-IV, p = 0.0275 in OSCC stage I-II and p = 0.0057 in post-treatment OSCC) in discriminating all OSCC patients from controls. Also LGALS3BP proved to be a highly significant indicator in OSCC stage I-II patients and high risk PMODs with p = 0.0008 and p = 0.0001, respectively; but was not discriminatory for late stage OSCC. Therefore, we can say that IL-1β and IL-8 are very good indicators of OSCC cases. On the other hand, LGALS3BP is a good indicator of early stage OSCC cases and PMODs but not for late stage OSCC cases.

**Table 1:** p-value: validation of saliva biomarkers in OSCC/ T1-T2/ T3-T4/ PMOD/ post-treatment subjects versus healthy control subjects.

Marker performance vs. control group	T1-T2	T3-T4	OSCC total	Post/under treatment	PMOD
IL-1β	0.0203*	<0.0001*	<0.0001*	0.0921	>0.9999
IL-8	0.0275*	0.0003*	0.0006*	0.0057*	0.2510
LGALS3BP	0.0008*	0.6360	0.0689	0.5905	0.0001*

*p < 0.05; p-values determined with Mann-Whitney test.

The performance of markers are usually reported in terms of sensitivity and specificity. The area under the receiver operating characteristics (ROC) curve (AUC) represents a relation between sensitivity and specificity. Thus it is an important measurement when reporting biomarker performance. It represents biomarker accuracy in a population. ROC is a probability curve and AUC represents degree or measure of separability. Higher the AUC, better the model is at distinguishing between patients with disease and no disease. An excellent model has AUC closer to 1 and a poor model has AUC closer to 0. For example, AUC = 0.7 means that there is a 70% chance that a biomarker will be able to distinguish between presence and absence of disease.

**Figure 2:**
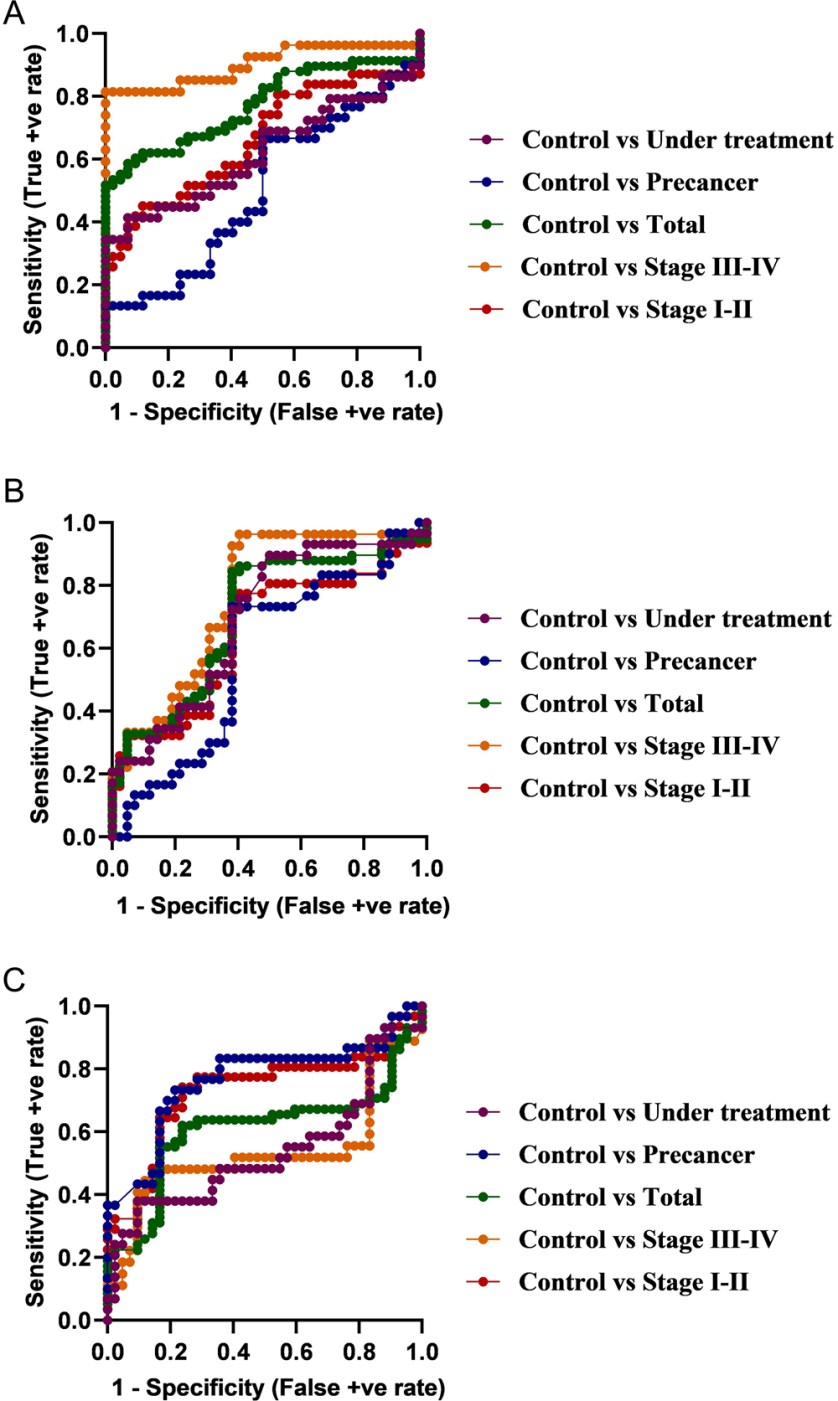
ROC (receiver operator characteristic) curve analysis for predictive power of salivary biomarkers, IL-1β (panel A), IL-8 (panel B) and LGALS3BP (panel C).

Figure 2 shows the ROC curves of IL-1β (panelA), IL-8 (panel B) and LGALS3BP (panel C). In ROC curve, the true positive rate (TPR) is plotted against the false positive rate (FPR). Since in all the three panels, the ROC curve is plotted above the diagonal (TPR = FPR), the markers prove to be good classifier of OSCC. In addition, the curve corresponding to late stage OSCC in Fig. 2A is the closest to the top left corner. This indicates that IL-1β (Fig. 2A) is the best performer of the three markers, especially in late stage OSCC (stage III-IV). In Fig. 2B all the curves except for that of PMODs is above the diagonal. Hence IL-8 seems to be a good performer for all stages OSCC cases. Figure 2C shows an interesting finding where the curves for early stage OSCC (stage I-II) and PMODs are to the top left corners, but that of late stage OSCC (stage III-IV) is below the diagonal. This suggests once again that LGALS3BP is a good performer for early stage OSCC and high risk PMODs, but not for late stage OSCC.

Table 2 shows the AUC values of the markers for each case category. It can be inferred that the AUC shows positive predictive power of all the three markers in all the case groups, as AUC was found to be greater than 0.5 in all the fields. However, highest predictive power was seen in case of OSCC stage III-IV. IL-1β had the strongest positive predictive value (AUC = 0.9017) followed by IL-8 (AUC = 0.7619) for late stage OSCC whereas LGALS3BP had strong positive predictive value for early stage OSCC (AUC = 0.7296) and PMODs (AUC = 0.7643). In fact, of the three protein markers studied LGALS3BP was the only discriminator between PMODs and controls.

**Table 2:** Area under roc curves (AUC).

Marker performance vs. control group	Area under ROC curve				
	Stage I-II	Stage III-IV	Total	Post/Under Treatment	PMOD
IL-1β	0.6598	0.9017*	0.7724*	0.6182	0.5000
IL-8	0.6517	0.7619*	0.7030*	0.6942	0.5798
LGALS3BP	0.7296*	0.5340	0.6069	0.5378	0.7643*

^*^AUC > 0.7.

An interesting observation in the post-treatment category of cases was noted. Thirteen patients (44.8%) were under chemotherapeutic medications postoperatively and presented with consistently increased levels of IL-8 but decreased levels of IL-1β. Among these postoperative follow-up cases, 8 patients presented with recurrence, confirmed on biopsy, where levels of both IL-1β and IL-8 were raised, but LGALS3BP levels were diminished. The patients with no recurrence as well as no medications showed relatively higher levels of LGALS3BP. Though these findings are not statistically significant here, it suggests that these protein markers may have the potential of predicting recurrences, if studied in more detail.

To be able to detect these biomarkers in saliva for use in screening methods, it is important that they have sufficient sensitivity and specificity. For all cancer patients, IL-1β had a high sensitivity of 71% and specificity of 59.5%. IL-8 and Mac-2BP presented with good sensitivity of 63.8% each. However these markers were not as sensitive or specific to detect pre-cancers or post-treatment cases. Table 3 presents the ROC curve analysis of salivary biomarkers in OSCC patients along with the maximum sensitivity and specificity values in this category. As evident from the table, the values suggest that all the three markers are excellent discriminators of OSCC from healthy control subjects.

**Table 3:** Receiver operator characteristic (ROC) curve analysis of OSCC associated salivary biomarkers.

Marker performance vs. control group	Maximum Sensitivity					Maximum Specificity				
	I-II	III-IV	Total	PMOD	Under Tt	I-II	III-IV	Total	PMOD	Under Tt
IL-1β	0.9677	0.9630	0.9828	0.9667	0.9655	1.000	1.000	1.000	1.000	1.000
IL-8	0.9677	0.9630	0.9828	1.000	0.9655	1.000	1.000	1.000	0.9762	1.000
LGALS3BP	0.9677	0.9630	0.9828	1.000	0.9655	1.000	1.000	1.000	1.000	1.000

## Discussion

Majority of oral cancers are OSCC, which when found early can have 80–90% survival rate. WHO has reported oral cancer as having the highest mortality ratios amongst other malignancies with a death rate at five years from diagnosis at 45%^31^. The high morbidity and mortality rate can therefore be due to the delayed diagnosis of the disease^32^. The commonly used clinical techniques such as biopsy, tissue processing and staining and cytology can be used only on small groups of patients who come to seek advice and treatment. Such methodology has certain limitations^33^. The aim of using salivary biomarkers for OSCC detection is that they can be useful for large scale screening purposes so that lesions can be detected easily without being expensive or invasive and can be used by non-trained individuals. For this saliva screening methods must have sufficient sensitivity and specificity. In the recent past a large array of accessible salivary biomarkers have been reported for OSCC detection^23,28,30,34–36^. These markers now needs to be validated to make them clinically applicable. This would further facilitate in development of point of care devices in order to provide easy to use diagnostic technology using salivary biomarkers^37–40^.

In this study, salivary biomarkers previously validated to be discriminatory of OSCC in other populations were selected for validation in the Indian population. Our results validate these previously found biomarkers as discriminatory between OSCC and controls, even in Indian cohort. This suggests that these OSCC biomarkers are independent of ethnic variations.

Of the three protein markers, IL-1β and IL-8, yielded significant predictive power for all OSCC cases with an AUC 0.7724 and 0.70301, respectively. For late stage OSCC, IL-1β and IL-8 had even higher predictive power with AUC 0.9017 and 0.7619, respectively. Unlike the other two, LGALS3BP did not yield such a strong predictive power for late stage OSCC. Instead, it yielded high predictive power for early stage OSCC and PMODs with AUC 0.7296 and 0.7643, respectively. The AUC values of IL-1β and IL-8 for the PMOD and post-treatment patients were poor, making them weak discriminators for PMODs or in post-treatment phases. However, LGALS3BP, with a significant AUC of 0.7643 in PMODs, makes it a good indicator to differentiate PMODs from non-suffering individuals. It can therefore be inferred that IL-1β and IL-8 may help us to distinguish the OSCC patients in large scale screening methods. In the same way LGALS3BP may help us in screening early stage OSCC and high risk PMODs from the general population. The sensitivity and specificity of individual markers were satisfactory to confirm them as discriminatory and to be used for screening methods. Our results regarding the biomarker levels are not identical but very similar to the previously found reports (13–22). In fact, our results are in agreement with an earlier finding where LGALS3BP was found to be a highly significant marker for early stage OSCC but was not discriminatory for late stage OSCC (16).

From our panel of salivary biomarkers, two are inflammatory markers (IL-1β and IL-8). Hence, their performance as oral cancer markers needs separate discussion. It has been previously reported that there is a significant difference for IL-8 levels in OSCC and periodontitis patients^12^. Our study confirms this discriminatory power as the case and control subjects which we selected were not suffering from oral inflammatory conditions yet presented with significantly elevated levels in all stages of OSCC. In a separate study, it was found that IL-8 could discriminate between PMODs and OSCC^22^ while in another study, such a fact could not be concluded^21^. The present study suggests that being strongly discriminatory for OSCC, IL-8 can be used to differentiate PMODs from OSCC cases. In addition, IL-1β was validated to be most discriminatory in accordance to previous findings^10,16,17,20^. While our findings strengthen the significance of salivary protein markers in OSCC detection, further studies are suggested using several biomarkers which takes into account the multifactorial pathologies of OSCC. At the same time, it is important to narrow down the choice of markers from the abundant array we presently have.

In this regards, statistical challenges are a significant factor in determining marker efficacy. Though AUC tells us about the biomarker performance in a population, it does not take into account, personal probability of developing disease. To estimate personal risk, in order to enhance screening results, PPV (positive predictive value) is the choice of statistical measure. PPV defines personal patient probability of developing a disease. The present study was a case-control design, hence, though it substantiates the predictive power of these markers, the PPV and NPV values are bound to be biased. This calls for a more prospective study design which follows the cohort forward in time and gives a better idea on the likelihood ratios (LR). The LR gives an idea of how well a biomarker can separate one signal from many. Biomarkers with higher LR would prove to be the best. LR’s can thus increase the efficiency of large scale screening procedures using fewer biomarkers^41,42^. Furthermore, it is imperative to validate the biomarker performance in multiple cohorts^43^.

## Conclusion

In this work, for the first time multiple salivary biomarkers were evaluated in Indian population, and the results potentiate the ability of IL-1β, IL-8 and LGALS3BP to be used as independent indicators of OSCC. Among the three protein markers (IL-1β, IL-8, and LGALS3BPBP) investigated, IL-1β and IL-8 were significantly elevated in all stages of OSCC patients, while LGALS3BP was significantly elevated specifically in early stage OSCCs and PMODs. Therefore, IL-1β and IL-8 proved to be significantly discriminatory between OSCC and controls, while LGALS3BP proved to be discriminatory between early and late stage OSCC as well as early stage OSCC + PMODs and controls. IL-1β and IL-8 were especially strong discriminators while LGALS3BP was a poor discriminator of late stage OSCC (stage III-IV). IL-1β had the most statistically significant discriminative power (AUC = 0.9017) in late stage OSCC followed by IL-8 (AUC = 0.7619). On the other hand, LGALS3BP was a stronger discriminator (AUC = 0.7296) of early stage OSCC than IL-1β and IL-8. The possibility of using biomarkers in saliva as a non-invasive tool in screening and diagnosis of oral cancer can thus be substantiated with the present study. Each participant included in the study had signed an informed consent.

## Supplementary information

Table S1 and Table S2.

Table S3.
