# Supplementary material for: Validation of Salivary Markers, IL-1β, IL-8 and Lgals3bp for Detection of Oral Squamous Cell Carcinoma in an Indian Population
Source: Sci Rep. 2020 Apr 30;10:7365. doi: 10.1038/s41598-020-64494-3 (PMC7192911; doi:10.1038/s41598-020-64494-3)
Supplement: Supplementary file 1 — Table S1 and Table S2. [file 41598_2020_64494_MOESM1_ESM.docx]

SUPPLEMENTARY INFORMATION

**VALIDATION OF SALIVARY MARKERS, IL-1β, IL-8 AND LGALS3BP FOR DETECTION OF ORAL SQUAMOUS CELL CARCINOMA IN AN INDIAN POPULATION**

Prerana Singh^1,*^, Jitendra K Verma^2^ and Jayant Kumar Singh^3^

1. Department of Oral Pathology, Maharana Pratap Dental College, Kanpur.
2. Department of Radiotherapy, J K Cancer Institute, Kanpur.
3. Department of Chemical Engineering, Indian Institute of Technology Kanpur.

*Author for all Correspondence:

Contact number: +91-9936335283

Email: [drprerana.singh@gmail.com](mailto:drprerana.singh@gmail.com)

Table S1: The inclusion and exclusion criteria used to select subjects.

|  | **INCLUSION CRITERIA** | **EXCLUSION CRITERIA** |
| --- | --- | --- |
|  | Presence of OSCC as confirmed after biopsy | Stomatitis, glossitis, cheilitis |
|  | Presence of PMOD such as oral submucous fibrosis, oral lichen planus and leukoplakia with degrees of dysplasia confirmed on biopsy | Gingivitis and/or periodontitis |
|  | Post-operative cases on follow-up | Apthous ulcers |
|  | OSCC patients under chemotherapy | Oral abscesses |
|  | Postoperative cases with recurrence |  |

Table S2: The epidemiological data

|  | Case subjects (117) | | | | | | | | | | Control subjects (42) | | | |
| --- | --- | --- | --- | --- | --- | --- | --- | --- | --- | --- | --- | --- | --- | --- |
| **Total number of subjects** | Early OSCC* | Late OSCC | | | Post/under treatment | | | | PMODs* | | 42 | | | |
|  | 31 | 27 | | | 29 | | | | 30 | |  |  |  |  |
| **Age** | | | | | | | | | | | | | | |
| Age (years)  (mean+/-SD) | 45.89 ± 12.36 | | | | | | | | | | 43.05 ± 10.40 | | | |
| Mean age (years)  (males) | 46.15±11.67 | | | | | | | | | | 43.18±11.02 | | | |
| Mean age (years)  (females) | 45.73±15.32 | | | | | | | | | | 42.63±8.31 | | | |
| **Sex** | | | | | | | | | | | | | | |
| Males (n ;%) | 95; 81.2% | | | | | | | | | | 34; 80.95% | | | |
| Females (n ;%) | 22;18.20% | | | | | | | | | | 8; 19.04% | | | |
| **Socioeconomic status** | >90%  (lower socioeconomic strata) | | | | | | | | | | >90%  (lower socioeconomic strata) | | | |
| **Habits** | No habit | Tobacco chewing | | Tobacco smoking | | | | Mixed habit pattern | | Others | Tobacco smoking/chewing | | | No habit |
| number | 5 | 60 | | 18 | | | | 37 | | 7 | 12 | | | 30 |
| % | 4.27 | 51.28 | | 15.38 | | | | 31.62 | | 5.98 | 28.57 | | | 71.42 |
| Duration of habit (years) | <10 | | 10-20 | | | | >20 | | | | <10 | 10-20 | >20 | |
| number | 21 | | 76 | | | | 15 | | | | 3 | 6 | 3 | |
| % | 18.75 | | 67.86 | | | | 13.39 | | | | 25 | 50 | 25 | |
| **Site of involvement** | | | | | | | | | | | | | | |
|  | Number | | | | | % | | | | |  | | | |
| BM* &/or LM* | 50 | | | | | 42.73 | | | | |  |  | |  |
| BM+GBS* | 24 | | | | | 20.51 | | | | |  |  | |  |
| Tongue | 18 | | | | | 15.38 | | | | |  |  | |  |
| Palate | 8 | | | | | 6.83 | | | | |  |  | |  |
| Extensive | 10 | | | | | 8.54 | | | | |  |  | |  |
| Others | 3 | | | | | 2.56 | | | | |  |  | |  |

*OSCC=Oral squamous cell carcinoma

PMODs= Premalignant oral disorders

BM= Buccal mucosa

LM= Labial mucosa

GBS= Gingivobuccal sulcus
