# Supplementary material for: Validation of Salivary Markers, IL-1β, IL-8 and Lgals3bp for Detection of Oral Squamous Cell Carcinoma in an Indian Population
Source: Sci Rep. 2020 Apr 30;10:7365. doi: 10.1038/s41598-020-64494-3 (PMC7192911; doi:10.1038/s41598-020-64494-3)
Supplement: Supplementary file 2 — Table S3. [file 41598_2020_64494_MOESM2_ESM.pdf]

SUPPLEMENTARY INFORMATION

VALIDATION OF SALIVARY MARKERS, IL-1β, IL-8 AND LGALS3BP FOR DETECTION OF ORAL SQUAMOUS CELL CARCINOMA IN AN INDIAN POPULATION

Prerana Singh(1),*, Jitendra K Verma(2) and Jayant Kumar Singh(3)
1. Department of Oral Pathology, Maharana Pratap Dental College, Kanpur.
2. Department of Radiotherapy, J K Cancer Institute, Kanpur.
3. Department of Chemical Engineering, Indian Institute of Technology Kanpur.

*Author for all Correspondence:
Contact number: +91-9936335283
Email: drprerana.singh@gmail.com

Table S3: Detailed epidemiological data.

S.No.	AGE	SEX	DIAGNOSIS	SITE	SIDE	OCCUPATION	HABIT	DURATION OF HABIT (years)	CLINICAL PRESENTATION	SIZE OF LESION	DURATION OF DISEASE	REGIONAL SPREAD	TNM SCORE	STAGE	H/P GRADE	INTERVENTION	SAMPLE LABEL
1	46	M	SCC	soft palate	left	tailor	bidi smoking	12	prolifearive	3x3cm	6months	absent	T2N0Mx		MDSCC	chemotherapy 2 d	p1
2	30	M	SCC	buccal mucosa	left	farmer	tobacco chewing bidi	5	ulceroindurative	>4cm	1 yr	LN +nt level I 2x2cm	T4aN1Mx			chemotherapy 2 d	p2
3	43	M	SCC	buccal mucosa+ gingiva	left	farmer	tobacco chewing	8	uncerative	>2.5cm	2 months	LN +nt level Ib and II	T4aN2bM0	stage Iva	WDSCC		p3
4	25	M	SCC	buccal mucosa+gingiva	left	unemplyed	tobacco chewing+cigarrete	15	ulceroproliferative	4X4cm	7months	absent	T4NoMx	stage Iva	WDSCC		p4
5	23	M	SCC	tongue(lateral border and base)	right	halwai/labour	gutkha+cigarrete	3	ulceroproliferative	3.5X2.5	6months	absent	T2NoMx	stage II	WDSCC		p5
6	60	M	SCC	buccal mucosa+gingiva	right	labour	tobacco chewing	5	ulcerative	3cm	6months	LN+nt levelIb+II	T4aN2bMx	stage IV			p6
7	40	M	SCC	tongue(anterior 2/3rd)+base of tongue	bilateral	labour	gutkha+tobacco chewing+bidi	15	ulceroproliferative	>4cm		LN+nt levelII	T4aN1Mx	stage Iva			p7
8	52	M	SCC	buccal mucosa+gingiva+rmt	left	tea seller	tobacco chewing	10	ulcerative	>2.5cm	2months	LN+nt levelIb+II	T4aN2bMx	stage IV	WDSCC	methotrexate	p8
9	27	M	SCC	buccal mucosa+gingiva	left	labour	gutkha+bidi	20	ulceroproliferative	3x2.5cm	5months	absent	T4N0Mx	stage IV	MDSCC	methotrexate	p9
10	40	M	LEUKOPLAKIA	buccal and labial mucosa	bilateral	farmer	tobacco chewing	2	nodular	3x3cm	2months	absent					p10
11	59	M	SCC	buccal mucosa+gingiva	left	labour	tobacco chewing+bidi	20	ulcerative	2x2cm	6months	absent	T2N0Mx	stageII	MDSCC		p12
12	55	M	SCC	soft palate		farmer	tobacco chewing	10	ulcerative	1cm	20days	absent	T1N0Mx	stage I	WDSCC		p13
13	50	M	SCC	buccal mucosa+gingiva	left	farmer	tobacco chewing	20	ulcerative	2cm	1 month	absent	T2N0Mx	stageII	WDSCC		p14
14	38	M	LEUKOPLAKIA+OSMF	buccal mucosa	left	labour	gutkha+bidi	5	pigmented homogeneous	1x0.5cm	1yr						p15
15	55	M	SCC	tongue(lat border)	left	labour	tobacco chewing	20	ulceroproliferative	0.5cm	2months	absent	T1N0Mx	stage I	WDSCC		p16
16	70	M	LEUKOPLAKIA	soft palate		businessman	gutkha+cigarrete	30	granular, nodular	2cm	6months						p17
17	35	F	SQUAMOUS PAPILLOMA	buccal mucosa	left	housewife	gutkha	15	proliferative, verrucuous	3cm	9months				papilloma		p18
18	35	M	SCC+OSMF	tongue tip	left	farmer	gutkha	15	proliferative, verrucuous	0.2cm	6months				verrucuous ca		p19
19	32	M	NO RECURRENCE	buccal mucosa	left	labour	no habit		ulcerative	3cm	2months	LN+nt levelI	T3N1M0	stage III	WDSCC	postoperative	p20
20	40	M	SCC	buccal mucosa+gingiva+rmt	left	labour	tobacco chewing	30	ulceroproliferative	>4cm	9 months	absent	T4NoMx	stage IV	WDSCC		p21
21	63	M	SCC	soft palate+post tongue+oropharyngeal wal	left	labour	tobaccochewing+gutkha+bidi	10	ulceroproliferative	3cm	2months	absent	T1N0Mx	stage I	WDSCC		p25
22	78	M	SCC	tongue+ext to neck	left	labour	bidi smoking	25	ulcerative	5x5cm	1yr	LN+nt level II	T4aN2cM1	stageIV	PDSCC		p27
23	25	M	SCC	buccal mucosa	right	unemployed	gutkha	10	ulcerative	4x5cm	4months	absent	T4aN0Mx	stageIV	WDSCC	methotrexate	p29
24	32	M	LEUKOPLAKIA	buccal mucosa	left	driver	gutkha	20	homogeneous	1x1cm	6months						p30
25	35	M	LEUKOPLAKIA	tongue		shopkeeper	gutkha+bidi	15	homogeneous	2x2cm	6months						p31
26	40	M	SCC	buccal mucosa+gingiva	left	labour	gutkha	10	ulcerative	1x1cm	3months	absent	T1N0M0	stage I	WDSCC		p33
27	54	M	SCC RECURRENT	buccal mucosa+rmt	left	labour	gutkha	20	ulceroproliferative	1x2cm	1month	LN+nt level I	T1N1M0	stage I	MDSCC	postoperative	p34
28	35	F	NO RECURRENCE	buccal mucosa+gingiva	left	tailor	tobacco chewing+bidi	15	ulcerative	2x3cm	1month	LN+nt	T2N1M0	stageII	WDSCC	postoperative	p37
29	58	M	SCC	buccal mucosa+gingiva	left	labour	gutkha+cigarrete	12	ulceroproliferative	2x2cm	2months	LN+nt level I&II	T4aN2aM0	stageIV	WDSCC		p38
30	45	M	SCC RECURRENT	tonsillar fossa		labour	gutkha+cigarrete	20	ulcerative	extensive	2months	LN +nt	T3N1M0	stageIV	WDSCC	surgery+radio+me	p40
31	50	M	SCC	buccal mucosa	right	labour	tobacco chewing	15	ulcerative	1x1cm	3months	absent	T1N0M0	stage I	WDSCC		p41
32	88	F	SCC	buccal mucosa+retromolar triangle	right	housewife	arecanut	20	ulcerative	3.5x1.5cm	4months	absent	T2N0M0	stageII	WDSCC		p42
33	65	F	CA +OSMF	tongue (lateral border)	right	housewife	tobacco chewing	40	ulcerative indurated	2x3cm	1month	absent	T4aN0M0	stageIV	WDSCC		p43
34	40	F	SCC RECURRENT	buccal mucosa	left	housewife	tobacco chewing	15	ulcerative indurated	2x3cm	6months	absent	T4aN0M0	stageIV	WDSCC	radiotherapy	p44
35	55	M	NO RECURRENCE	angle of mouth	leftCA+righ	farmer	tobacco chewing+bidi	40	verrucuous lesion	2x3cm	6months	absent	T2N0M0	stageII	WDSCC	postoperative	p45
36	50	M	CA +OSMF	hard & soft palate		labour at tobacco f	tobacco toothpowder	15	ulcerative	2x3cm	1yr	absent	T2N0M0	stageII	WDSCC	methotrexate	p46
37	70	M	OLP	buccal mucosa	bilateral	businessman	cigarrete	55	erythematous+striae	1x3cm	3-4yrs						p48
38	32	F	LEUKOPLAKIA	buccal mucosa+tongue	bilateral	housewife	gutkha+tobacco dentrifice	1	diffuse	extensive	6months						p50
39	62	M	LEUKOPLAKIA	lower labial mucosa	right	librarian	bidi smoking	40	homogeneous	1.5x1cm	1 year						p51
40	59	M	SCC+LEUKOPLAKIA	soft palate+retromolartriangle+lower lip	right	labour	tobacco chewing	15	ulcerative	0.5X1cm	1 month	absent	T1N0M0	stage I	WDSCC		p53
41	43	M	SCC	buccal mucosa+vestibule	left	labour	tobacco chewing	20	ulcerative	1x2cm	4 months	absent	T1N0M0	stage I	WDSCC		p54
42	58	M	SCC	soft palate+retromolar triangle	central	businessman	tobacco chewing+bidi	20	ulcerative	2x2cm	2months	absent	T1N0M0	stage I	WDSCC		p55
43	40	M	SCC	angle of mouth	right	labour	gutkha+bidi	10	ulceroproliferative	3.5x1.5cm	6months	LN +nt	T2N1M2	stageIII	WDSCC		p56
44	33	M	OSMF	buccal mucosa	bilateral	electrician	gutkha	7	blanching	extensive	5 yrs						p58
45	32	M	SCC	buccal mucosa+alveolus+gingivobuccal sul	left	labour	tobacco chewing+bidi	7	ulceroproliferative	>4cm	1 month	LN +nt	T4aN2aMo	stage IV	WDSCC		P2
46	56	M	LEUKOPLAKIA	buccal mucosa retromolar region	bilateral	labour	pan	10	homogeneous	1x1cm	1 month						P3
47	32	M	LEUKOPLAKIA	buccal mucosa near angle of mouth	left	labour	bidi smoking	30	homogeneous	1x1cm	7 yrs						P4
48	52	M	SCC	buccal mucosa+retromolar region	right	labour	tobacco chewing	2	ulceroproliferative	>4cm	2 months	LN +nt	T4aN2bMx	stage IV	WDSCC		P5
49	40	M	SCC	tongue (lateral border)	right	labour	tobacco chewing	4	verrucuous lesion	2x2.5cm	2 months	LN +nt	T2N1M0	stage II	WDSCC		P6
50	50	M	SCC	supraglottis	right	farmer	tobacco chewing+smoking bidi	30	ulcerative	5x4 cm	3months	LN +nt	T3N3Mx	stageIII	WDSCC		P7
51	45	M	SCC	buccal mucosa	left	labour	tobacco chewing`	30	extraoral perforation	extensive	4 months	LN +nt	T4aN2bMx	stage IV	WDSCC		P8
52	47	M	NO RECURRENCE	buccal mucosa	right	businessman	tobacco+gutkha	10	facial swelling+reduced mouth c	4x4cm	6 months	LN +nt	TxNxMx	stage IV	WDSCC	postoperative	P10
53	41	M	SCC	buccal mucosa	right	labour	tobacco chewing bidi	10	ulcerative	3x3cm	1 year	LN +nt	T4aN2bM0	stage IV	MDSCC		P12

54	52	M	OLP	buccal mucosa	bilateral	shopkeeper	tobacco chewing	10	white patch with striae	1x0.5cm	2 years						P13
55	44	M	OSMF	buccal mucosa	bilateral	driver	panmasala	20	blanching	extensive	5 years						P14
56	48	M	LEUKOPLAKIA	buccal mucosa	bilateral	framer	tobacco+bidi	20	homogeneous	2x2cm	6 months						P15
57	34	M	SCC	buccal mucosa(posterioe buccal space)	right	farmer	tobacco chewing	5	ulceroproliferative	1x1cm	1 month	LN +nt	T3N1M0	stage III	WDSCC		P17
58	37	M	LEUKOPLAKIA	buccal mucosa	bilateral	labour	tobacco chewing	2	homogeneous	2x2.5cm	2years						P18
59	42	M	SCC	buccal mucosa	right	farmer	tobacco chewing	4	ulceroproliferative	3x3cm	6months	LN +nt	T3N1M0	stage III	WDSCC		P19
60	46	M	SCC RECURRENT	tongue (lateral border)	right	labour	tobacco chewing	10	ulcerative	1x1cm	1month	LN +nt	T2N3bMx	stage III	WDSCC	postoperative	P20
61	60	F	SCC	tongue (lateral border)	right	housewife	tobacco chewing	15	ulcerative	3x3cm	2 months	absent	T4aN0Mx	stage IV	WDSCC		P21
62	67	M	SCC RECURRENT	enlarged node	right	farmer	no habit				1month	LN +nt				postoperative	P23
63	40	F	SCC	tongue (lateral border)	left	housewife	no habit		ulcerative	0.5x0.5cm	1 month	absent	T1N0M0	stage I	WDSCC		P24
64	47	M	SCC RECURRENT	tongue (lateral border)	right	labour	tobacco chewing	15	ulcerative	1x1cm	2months	LN +nt	T2N0M0	stage II	MDSCC	postoperative	P31
65	63	M	SCC	buccal mucosa+angle of mouth+alveolus+re	right	businessman	panmasala	20	ulcerative	>4cm	6 months	LN +nt	T4aN1M0	stage IV	WDSCC		P32
66	59	M	SCC RECURRENT	buccal mucosa+gingivobuccal sulcus	right	farmer	tobacco chewing	30	reduced mouth opening	cannot be assesse	2months	LN +nt	T3N2M0	stage III	WDSCC	postoperative	P34
67	69	F	SCC	base of tongue	right	housewife	tobacco tooth powder	10	ulceroproliferative	>4cm	6 months	LN +nt	T4a N2bMx	stage IV	MDSCC		P35
68	50	M	SCC	buccal mucosa+gingivobuccal sulcus+floor	left	carpenter	tobacco chewing	10	ulceroproliferative	>4cm	1 year	LN +nt	T4a N2bMx	stage IV	WDSCC		P36
69	45	M	SCC	angle of mouth+buccal mucosa+gingivobuc	right	farmer	tobacco chewing	20	ulceroproliferative	>4cm	2 months	LN +nt	T3N1Mx	stage III	WDSCC		P37
70	35	M	SCC RECURRENT	buccal mucosa	right	unemployed	tobacco chewing	15	facial swelling+reduced mouth c	2x3cm	1 month	LN +nt	T2N2Mx	stage II	WDSCC	postoperative	P38
71	35	M	SCC	buccal mucosa	left	labour	tobacco chewing+smoking bidi	20	ulcerative	2x2.5cm	2 months	absent	T2N0M0	stage II	WDSCC		P39
72	43	M	LEUKOPLAKIA	buccal mucosa	right	priest	tobacco+gutkha	20	homogeneous	1x1cm							P40
73	50	M	SCC	buccal mucosa+gingivobuccal sulcus	left	unemployed	tobacco chewing	20	swelling in submandibular regio	2x2cm	2 months	LN +nt	T4aN3bM0	stage IV	WDSCC	methotrexate	P41
74	60	F	SCC RECURRENT	tongue (lateral border)	left	farmer	tobacco chewing	30	ulcerative	2x2cm	1 month	LN +nt	T2N2Mx	stage II	WDSCC	postoperative	P42
75	51	M	SCC+OSMF	hard palate	left	tailor	tobaccochewing+bidi smoking	20	ulcerative	1.5x2cm	6 months	LN +nt	T4bN2bMx	stage IV	WDSCC	methotrexate	P44
76	47	M	SCC	buccal mucosa	right	shopkeeper	tobacco chewing	16	ulcertaive	1.5x1.5cm	2 months	absent	T1N0M0	stage I	WDSCC		P45
77	44	M	LEUKOPLAKIA	buccal mucosa	right	teacher	tobacco chewing	10	homogeneous	1x1cm	6 months						P46
78	38	F	NO RECURRENCE			housewife	panmasala	10								postoperative	P47
79	46	F	SCC	hard palate+soft palate	right	housewife	tobacco chewing	5	reduced mouth opening	MO=1cm	1 year	LN +nt	T4aN1M0	stage IV	WDSCC	methotrexate	P48
80	34	F	LEUKOPLAKIA	hard palate+tongue+buccal mucosa	right	housewife	gul manjan	8	greyish white patch	multiple +extensiv	8 months						P49
81	50	M	SCC	buccal mucosa	right	labour	tobacco chewing	15	ulcerative	2.5x2cm	6 months	absent	T2N0M0	stage II	WDSCC		P50
82	53	M	LEUKOPLAKIA	buccal mucosa	bilateral	clerk	panmasala	10	homogeneous pigmented	1x2cm	4 months						P51
83	45	F	SCC	buccal mucosa	left	housewife	tobacco chewing	10	ulcerative	3x3cm	6 months	absent	T2N0M0	stage II	WDSCC		P52
84	39	M	SCC	buccal mucosa	left	labour	tobacco chewing	10	ulcerative	0.5X1cm	3months	absent	T1N0M0	stage I	WDSCC		P53
85	43	F	SCC RECURRENT	buccal mucosa	left	housewife	no habit		ulceroproliferative+RMO	cannot be assesse	1 month	absent	T3N0Mx	stage III	WDSCC	postoperative	P55
86	32	M	SCC	buccal mucosa	right	unemployed	tobacco chewing	15	ulcerative	>4cm	2 years	absent	T3N0Mx	stage II	WDSCC		P56
87	70	M	LEUKOPLAKIA	tongue (lateral border)	bilateral	retired	panmasala	30	homogeneous	extensive	5 months						P58
88	39	M	SCC	buccal mucosa	right	farmer	gutkha+bidi	10	ulcerative	2.5x2cm	1 year	absent	T2N0M0	stage II	WDSCC		P59
89	30	F	LEUKOPLAKIA	buccal mucosa	left	labour	panmasala	20	homogeneous	1x1cm	1year						P60
90	45	F	SCC	buccal mucosa+angle of mouth	right	housewife	tobacco chewing	16	ulceroproliferative	>4cm	5 months	LN +nt	T4aN0Mx	stage IV	WDSCC		P61
91	66	M	SCC	buccal mucosa+alveolus+retromolar triang	left	farmer	bidi smoking	20	ulcerative	2x3cm	1 year	LN +nt	T3N1M0	stage III	WDSCC		P62
92	57	M	NO RECURRENCE			driver	tobacco chewing	15								postoperative	P63
93	38	M	SCC	buccal mucosa+gingivobuccal sulcus	right	shopkeeper	mixed	10	ulceroproliferative	2x2cm	1 year	LN +nt	T4aN1Mx	stage IV	MDSCC	chemotherapy (6	P64
94	31	M	SCC	buccal mucosa	left	unemployed	tobacco chewing	10	white vegetative growth	1x1cm	1 month	absent	T1N0Mx	stage I	WDSCC		P65
95	37	M	LEUKOPLAKIA	buccal mucosa	right	labour	cigarrette	10	homogeneous	0.5x1cm	2 months						P67
96	48	M	NO RECURRENCE			farmer	bidi	20	MO=0cm							postoperative	P68
97	50	F	SCC	buccal mucosa	right	housewife	mixed	10	ulceroproliferative	>2.5x1.5cm	1.5months	absent	T2N0M0	stageII	MDSCC		P69
98	45	M	LEUKOPLAKIA	buccal mucosa	bilateral	officer	mixed	5	homogeneous	0.5x0.5cm	6 months						P70
99	37	M	SCC	tongue (lateral border)	left	driver	tobacco chewing	20	ulcerative	2.5x2.5cm	2 months	absent	T2N0M0	stage II	WDSCC		P71
100	50	F	SCC	tongue (lateral border)	left	housewife	tobacco chewing	30	ulcerative	1x1cm	2 months	absent	T1N0M0	stage II	WDSCC		P72
101	41	M	LEUKOPLAKIA	buccal mucosa	left	driver	tobacco chewing	20	homogeneous	0.5x0.5cm	1 month						P73
102	68	M	SCC	lower lip	right	farmer	tobacco+bidi	20	white vegetative growth	2x3cm	3months	absent	T2N0M0	stage II	WDSCC		P74
103	60	M	NO RECURRENCE			labour	tobacco chewing	7								postoperative	P75
104	23	M	LEUKOPLAKIA+OSMF	buccal mucosa	left	student	tobacco chewing	2	homogeneous patch+blanching	1x1cm	4months						P76
105	40	M	SCC	gingivobuccal sulcus mandibular	left	carpenter	tobacco+gutkha	10	ulcerative	2x2cm	8 months	LN +nt	T2N1M0	stage II	WDSCC		P77
106	26	F	SCC	Floor of mouth	right	housewife	no habit		erythematous	2x1cm	1.5yrs	absent	T1N0Mx	stage I	MDSCC		P78
107	61	M	SCC	cannot be assessed		labour	bidi smoking	30	cannot be assessed		2 yrs	LN +nt	TxNxMx	stage IV	metastatic SCC		P79
108	50	M	SCC	buccal mucosa	right	milkman	tobacco chewing	7	ulcerative	2x2.5cm	6 months	LN +nt	T2N1M0	stage II	WDSCC		P81
109	38	M	SCC	tongue (lateral border)	bilateral	shopkeeper	tobacco+gutkha	10	ulcerative	1.5x2.5cm	8 months	LN +nt	T2N2cM0	stage II	WDSCC		P82
110	37	M	SCC	gingivobuccal sulcus mandibular	right	labour	gutkha	13	ulcerative	1x3cm	2months	absent	T1N0M0	stage I	WDSCC		P84
111	39	M	SCC	Loweralveolus+gingivobuccal sulcus+retror	right	farmer	tobacco+bidi smoking	10	ulcerative	3x2cm	6 months	LN +nt	T4aN1M0	stage IV	Keratinising SCC		P86
112	45	M	SCC	cheek+angle of mouth+upper lip	left	trader	tobacco chewing	10	ulceroproliferative, hard	4x4cm	5 months	LN +nt	T3N1M0	stageIII	sarcomatoid SCC		P87
113	38	F	LEUKOPLAKIA	soft palate		housewife	supari	8	homogeneous pigmented	2x2cm	3 yrs						P88
114	42	M	SCC	buccal mucosa	right	cleaner	tobacco chewing	10	ulcerative	2x2cm	7 months	absent	T1N0M0	stage I	WDSCC		P89
115	38	M	SCC	buccal mucosa+retromolar triangle	left	labour	tobacco chewing	12	facial swelling+reduced mouth c	cannot be assesse	4months	LN +nt	T4aN2bM0	stage IV	WDSCC	chemotherapy	P90
116	45	M	LEUKOPLAKIA+OSMF	buccal mucosa	bilateral	cabdriver	tobacco chewing	14	homogeneous patch and blanch	multiple +extensiv	1 year						P91
	27	F	ERYTHROPLAKIA	buccal mucosa	right	housewife	tobacco chewing	13	erythematous	1x1cm	2months						P92
	AGE	SEX	HABIT	OCCUPATION													SAMPLE LABEL
1	28	M	smoker	student				5									c1
2	54	M	smoker	farmer				25									c2
3	53	M	non smoker	farmer													c3
4	52	M	non smoker	clerk													c5
5	47	M	non smoker	labour													c6
6	46	F	non smoker	housewife													c7

7	45	M	chewer	sweeper				20									c8
8	45	F	non smoker	housewife													c9
9	42	F	non smoker	housewife													c10
10	26	F	non smoker	housewife													c12
11	61	M	chewer	farmer				30									c14
12	37	M	non smoker	farmer													c15
13	54	F	non smoker	housewife													c17
14	31	M	non smoker	labour													c19
15	54	M	non smoker	farmer													c20
16	49	F	non smoker	housewife													c21
17	40	F	non smoker	housewife													c22
18	60	M	non smoker	labour													c23
19	30	M	chewer	priest				5									c24
20	59	M	chewer	farmer				20									c26
21	38	M	non smoker	trader													c27
22	39	M	non smoker	labour													c28
23	29	M	smoker	labour				8									c29
24	42	M	non smoker	labour													c30
25	53	M	chewer	shopkeeper				15									c31
26	36	M	non smoker	milkman													c32
27	40	M	chewer	labour				15									c33
28	25	M	non smoker	student													c34
29	50	M	smoker	carpenter				25									c36
30	43	M	non smoker	farmer													c37
31	39	F	non smoker	housewife													c38
32	68	M	non smoker	farmer													c39
33	45	M	non smoker	businessman													c40
34	51	M	non smoker	driver													c41
35	28	M	non smoker	cleaner													c42
36	41	M	chewer	tailor				15									C93
37	41	M	chewer+smoker	farmer				15									C95
38	33	M	non smoker	clerk													C101
39	39	M	non smoker	shopkeeper													C103
40	31	M	non smoker	labour													C105
41	35	M	non smoker	driver													C106
	50	M	non smoker	officer													C108
